# Enrichment of Nutmeg Essential Oil from Oil-in-Water Emulsions with PAN-Based Membranes

**DOI:** 10.3390/membranes14050097

**Published:** 2024-04-25

**Authors:** Huilan Yin, Haoyu Zhang, Jiaoyang Cui, Qianlian Wu, Linlin Huang, Jiaoyue Qiu, Xin Zhang, Yanyu Xiang, Bo Li, Hongbo Liu, Zhishu Tang, Yue Zhang, Huaxu Zhu

**Affiliations:** 1 Jiangsu Botanical Medicine Refinement Engineering Research Center, Nanjing University of Chinese Medicine, Nanjing 210023, China; yinhui357@163.com (H.Y.); haoyu09988@163.com (H.Z.); cjy101520@163.com (J.C.); wql961114@163.com (Q.W.); 17318229765@163.com (L.H.); 18851092313@163.com (J.Q.); zxin1163@163.com (X.Z.); xyy990815@163.com (Y.X.); boli@njucm.edu.cn (B.L.); 2 The First Clinical Medical College, Nanjing University of Chinese Medicine, Nanjing 210023, China; 3 Shaanxi Collaborative Innovation Center of Chinese Medicinal Resources Industrialization, Shaanxi University of Chinese Medicine, Xianyang 712046, China; 15319084280@126.com (H.L.); tzs6565@163.com (Z.T.)

**Keywords:** oil/water separation, stable polyacrylonitrile membrane, essential oil, non-destructive separation

## Abstract

This study used polyacrylonitrile (PAN) and heat-treated polyacrylonitrile (H-PAN) membranes to enrich nutmeg essential oils, which have more complex compositions compared with common oils. The oil rejection rate of the H-PAN membrane was higher than that of the PAN membrane for different oil concentrations of nutmeg essential oil-in-water emulsions. After heat treatment, the H-PAN membrane showed a smaller pore size, narrower pore size distribution, a rougher surface, higher hydrophilicity, and higher oleophobicity. According to the GC-MS results, the similarities of the essential oils enriched by the PAN and H-PAN membranes to those obtained by steam distillation (SD) were 0.988 and 0.990, respectively. In addition, these two membranes also exhibited higher essential oil rejection for Bupleuri Radix, Magnolia Officinalis Cortex, Caryophylli Flos, and Cinnamomi Cortex essential oil-in-water emulsions. This work could provide a reference for membrane technology for the non-destructive separation of oil with complex components from oil-in-water emulsions.

## 1. Introduction

Essential oils are significant bioactive components found in aromatic traditional Chinese medicine (TCM) [1]. They exhibit a wide range of biological activities, including antibacterial, antiviral, anti-inflammatory, and antioxidant properties [2,3]. Essential oil is the primary bioactive compound in nutmeg (*Myristica fragrans* Houtt.), which belongs to the Myristica genus within the nutmeg family [4]. The essential oil from nutmeg (EON) possesses bactericidal and analgesic properties, making it a valuable resource for treating conditions such as rheumatism, diarrhea, cholera, intestinal diseases, and stomach spasms [5,6]. Moreover, EON is an essential commodity employed as a flavoring agent in the food, pharmaceutical, and cosmetic industries [7,8]. Consequently, there is a compelling need to develop efficient methods for obtaining the essential oil.

The essential oils used in TCM are known for their volatilities and instabilities, so they are susceptible to decomposition when exposed to light, heat, and air [9]. These specific attributes contribute to the challenges of extracting essential oils effectively. Currently, the prevalent methods used for extracting essential oils for TCM include distillation, solvent extraction, gas extraction, and supercritical fluid extraction [10]. At present, steam distillation (SD) is often used because it does not require organic solvents and preserves the integrity of the components. During the extraction, in addition to obtaining pure essential oil, many aromatic aqueous solutions with dispersed oil, emulsified oil, and dissolved oil are produced [11]. Consequently, there is an urgent need to develop an efficient, environmentally friendly, and safe technology for separating essential oils from water to address these challenges effectively [12,13].

Membrane separation is an efficient technology for oil/water separation, owing to its high efficiency, low energy consumption, simple operation processes, and minimal secondary pollution [14,15,16,17]. S.K. Gopika et al. [18] utilized nanofiltration to separate essential oils from ginger butter extracted with n-hexane, which effectively obtained high-quality turmeric oil with improved stability. C. Du et al. [19] prepared organic composite membranes and used them for pervaporation to extract valuable essential organic compounds from dilute aqueous solutions of Perilla frutescens. H. Xiao et al. [20] combined ceramic membrane microfiltration and poly (dimethyl siloxane)/poly (vinylidene fluoride) composite membrane pervaporation for separation of the thioether compounds in garlic oil. These results indicate that membrane separation technology has great potential for the separation and application of essential oils in TCM [21].

Polyacrylonitrile (PAN) membranes are widely used in industrial oil/water separations and have good chemical properties, thermal stabilities, and solvent resistance [22,23,24,25,26]. D. Teng et al. [27] fabricated SiO_2_/zein/PAN fiber membranes, which exhibited high oil/water separation efficiencies and separation fluxes. N. Xue et al. [28] used a collagen fiber membrane (CFM) as a multifunctional carrier and electrospun a PAN layer in situ to prepare a PAN/CFM composite membrane with an ultrahigh separation flux and high fouling resistance for preparing oil-in-water lotions.

Unlike most of the oils reported, the essential oil components of TCM are more complex [29,30,31]. According to their chemical structures, essential oils can be divided into terpenoids, including monoterpenes and sesquiterpenes, and their oxygen-containing derivatives, such as α-pinene β-pinene and limonene; aromatic compounds, such as eugenol and nutmeg ether; and aliphatic compounds, such as houttuynin and n-nonyl alcohol [32]. There are components in these complex organic compounds with similar polarity to PAN, resulting in damage to the PAN membrane. However, there is still limited research on the application of organic membranes, especially PAN membranes, in the enrichment of essential oils, and the membrane separation process still needs further investigation.

In this work, a heat-treated polyacrylonitrile (H-PAN) membrane was fabricated by annealing under inert gas to enhance the enrichment of EON. The investigation involved a comprehensive analysis of changes in the pore size distribution, microstructure, surface chemical structure, and wetting behavior of the PAN and H-PAN membranes. The enrichment of EON with the two respective membranes was compared by considering the oil rejection rates, fluxes, pollution models, and oil quality. It was extended to other TCMs used in research, such as Bupleuri Radix (BR), Magnolia Officinalis Cortex (MOC), Caryophylli Flos (CF), and Cinnamomi Cortex (CC). This research provides insight into PAN-based membranes with potential application in the enrichment of essential oils for TCM.

## 2. Materials and Methods

### 2.1. Materials

A PAN ultrafiltration membrane (molecular weight cut off 50 kDa) was purchased from RisingSun Membrane Technology, Beijing, China. The nutmeg herb was purchased from Jiangsu Chengkai Chinese Medicine Co., Ltd., Huaian, Jiangsu, China. Bupleuri Radix, Magnolia Officinalis Cortex, Caryophylli Flos, and Cinnamomi Cortex were purchased from Shaanxi Sciendan Pharmaceutical Co., Ltd., Tongchuan, Shaanxi, China. Sulfuric acid was purchased from Sinopharm Group Chemical Reagent, Shanghai, China. Ethyl acetate and diiodomethane were purchased from Aladdin, Shanghai, China. Chemical Oxygen Demand (COD) oxidant and COD catalyzer were purchased by Lvyu, Qingdao, Shandong, China. The COD was determined via a multifunctional water quality test (LY-4DB, Lvyu, Qingdao, Shandong, China). Deionized water was prepared with a water purification system (EPED-E2-20TS, Yipu Yida, Nanjing, Jiangsu, China).

### 2.2. Fabrication of H-PAN Membrane

First, the PAN membrane was soaked in deionized water for 24 h, and the water was changed three times to wash off the protective surface coating of the membrane. Then, the sample was dried and placed in a tube furnace. The temperature was raised to 200 °C at 2 °C/min and maintained at 200 °C for 1.5 h under argon protection.

### 2.3. Characterization

The thermogravimetric properties of the PAN polymer membrane were analyzed with a thermogravimetric analyzer (TG, STA 8000, PerkinElmer, Waltham, MA, USA). The chemical structure changes of the PAN polymer membrane during heat treatment were analyzed with a Fourier transform infrared spectrometer (FTIR, Nicolet iS20, Thermo Scientific, Waltham, MA, USA) and X-ray photoelectron spectroscopy (XPS, K-Alpha, Thermo Scientific, Waltham, MA, America). The surface morphology of membranes was observed by field-emission scanning electron microscopy (FE-SEM, Regulus-8100, Tokyo, Japan). Pore size was calculated with Brunauer–Emmett–Teller (BET, ASAP2460, Micromeritics, Norcross, GA, USA). The surface microstructure and roughness were observed with an atomic force microscope (AFM, Dimension ICON, Bruker, Billerica, MA, USA). Contact angle measurement (CA, DSA100, Kruss, Hamburg, Germany) was employed to characterize the wettability of the membranes. Optical photographs were taken with a biomicroscope (BX35, Olympus, Tokyo, Japan). The size distribution was measured with a nanoparticle size analyzer (Zetasizer Nano ZS90, Malvern Panalytical, Malvern, UK). The compositions of EON were analyzed by gas chromatography-mass spectrometry (GC-MS, TQ8050NX, Shimadzu, Kyoto, Japan).

### 2.4. Preparation of Essential Oil-in-Water Emulsion

**Figure 1:**
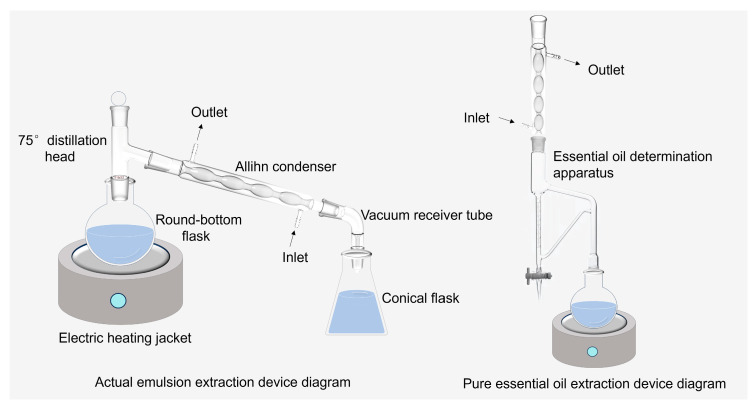
The diagrams of the actual emulsion and pure essential oil extraction devices.

An appropriate amount of nutmeg was accurately weighed. Then, it was crushed, passed through a No. 1 sieve, and placed in a round-bottom flask. Fourteen volumes of water were added to the herbs and soaked for 1 h. The extraction device is shown in Figure 1. The distillate was collected by SD to obtain an essential oil-in-water emulsion. Pure essential oil was extracted according to method A, Part IV “Determination Method of Essential Oil” in the Chinese Pharmacopoeia 2020. After cooling and stratification, the essential oil was collected and stored for use in brown bottles at a low temperature. Different volumes of pure EON extracted in the previous step were taken, and deionized water was added while stirring for 2 h at 500 r/min to obtain nutmeg essential oil-in-water emulsions with different concentrations. The BR, MOC, CF, and CC essential oil-in-water emulsions were collected by SD with 10 times the volume of water.

### 2.5. Oil/Water Separation

**Figure 2:**
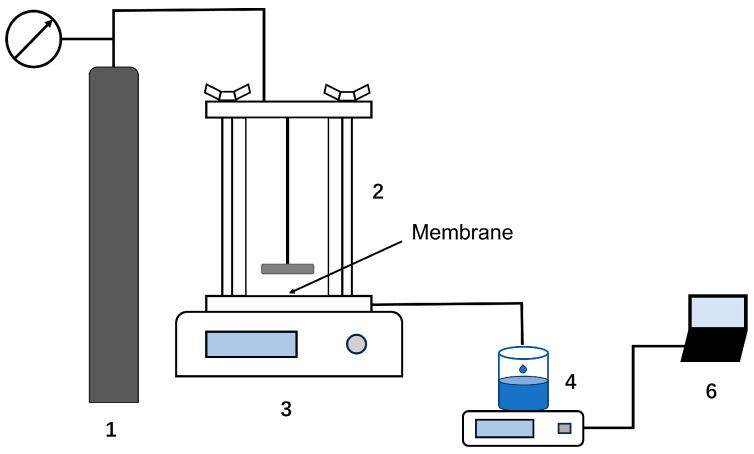
The schematic diagram of the ultrafiltration membrane separation device. (1) Nitrogen bottle; (2) ultrafiltration cup; (3) magnetic stirrer; (4) permeate vessel; (5) electronic balance; (6) computer.

The permeation performance evaluation was conducted using ultrafiltration equipment (Millipore, XFUF07601, USA), which is schematically presented in Figure 2. The effective area of the membranes was 34.2 cm^2^. The applied transmembrane pressure was constant at 0.2 MPa, and the stirring speed was 200 r·min^−1^ at room temperature. The permeate flux was calculated as the following equation:

(1) $$J=\frac{V}{\Delta PAt}$$

where *J* is the flux (L/(m^2^·h^−1^·MPa^−1^), *V* is the filtrate volume (L), *t* is per unit time (h), *A* is the effective area (m^2^), and Δ*P* is the applied trans-membrane pressure (MPa).

### 2.6. Membrane Fouling Mechanism

Hermia [33] developed four classical models of dead-end filtration based on Darcy’s law to explain the membrane fouling mechanisms: complete blocking, intermediate blocking, standard blocking, and cake filtration models [34,35,36,37]. The equation can be written as

(2) $$\frac{d^{2}t}{dV^{2}}={K(\frac{dt}{dV})}^{n}$$

where *t* is the filtration time (h), *V* is the cumulative filtration volume (L), *K* is the fouling constant, and *n* is the parameter that determines the type of membrane contamination [38]. The corresponding blocking models are presented in Table 1.

### 2.7. Determination of Rejection Rate

The permeating solution obtained through the membrane was determined by the classical potassium dichromate method. A 3 mL sample was added with 1 mL COD oxidant and 5 mL reducing agent, then digested for 10 min. Then, 3 mL of distilled water was added to the above solution. After cooling to room temperature, it was used to determine the COD value of organic matter in the sample. The calculation formula for the oil rejection rate is as follows:

(3) $$R=\frac{COD_{0}-COD_{1}}{COD_{0}}\times 100\%$$

where COD_0_ is the initial oil-in-water emulsion COD value, and COD_1_ is the permeate COD value.

### 2.8. GC-MS Analysis of the Enriched EON

The quality of EON separated by PAN and H-PAN membranes and the SD method was evaluated by GC-MS [39]. First, the enriched oil was diluted with ethyl acetate. The conditions for GC-MS chromatographic analysis were as follows: SH-Rxi-5Sil MS capillary column (30 m × 0.25 μm, 0.25 mm); helium as carrier gas (volume fraction was 99.999%); volumetric flow rate (1.5 mL/min); split ratio (30:1). The heating program is shown in Table 2. The spectrum library was searched through the Nist 20 MS Search data system of the chemistry workstation to confirm the chemical composition of the essential oil of the sample under test. The area normalization method was used to measure the relative percentage content of each component.

## 3. Results

### 3.1. Characterization

**Figure 3:**
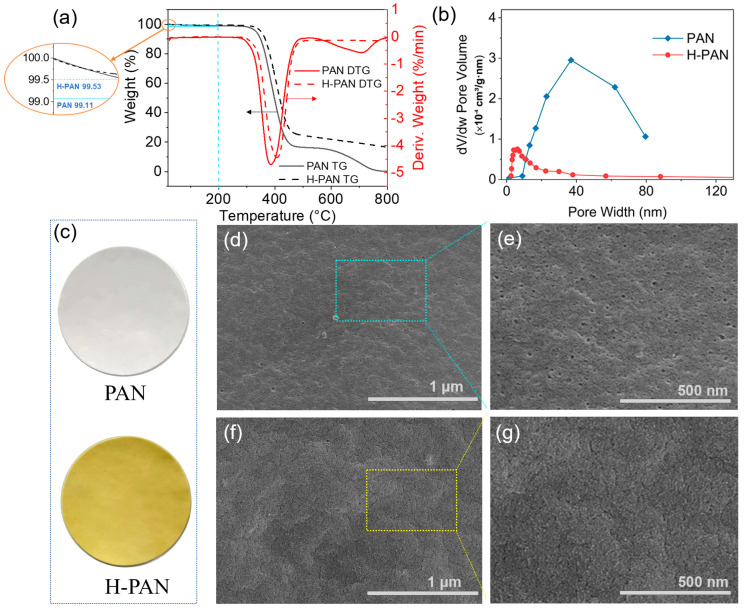
(**a**) The TG and DTG curves of PAN and H-PAN membranes; (**b**) the pore size distribution of PAN and H-PAN membranes; (**c**) the photographs of PAN and H-PAN membranes; the SEM images of the PAN (**d**,**e**) and H-PAN (**f**,**g**) membranes.

Essential oils contain abundant organic compounds that are corrosive to polymer materials. To broaden the application of PAN membranes for essential oil-in-water emulsions, heat treatment was used to modify the membranes. TG and DTG were used to observe the thermogravimetric properties of the PAN membrane and evaluate its stability regarding the heat treatment temperatures [40,41]. Figure 3a shows the weight changes and weight change rates for the PAN and H-PAN membranes with increasing temperatures from 20 to 800 °C in an argon atmosphere. The results indicated that the properties of the PAN membrane remain stable after heat treatment. The PAN membrane exhibited obvious weight losses at 300–450 °C and 600–750 °C under an argon atmosphere. At 750 °C, the PAN membrane was completely decomposed without any change in weight. The derivative thermogravimetry (DTG) curve showed that the weight change rate reached its maximum near 400 °C, indicating that the structure of PAN undergoes drastic changes around this temperature. However, the weight of the H-PAN membrane no longer changes after 600 °C. When the temperature was between 20 and 300 °C, the weight of the PAN membrane did not change significantly. When the temperature was 200 °C, the remaining weight of the PAN membrane was 99.11%, and the remaining weight of the H-PAN membrane was 99.53%, which was higher than that of the PAN membrane (as enlarged in Figure 3a). The weight loss may be caused by the transformation of a small number of functional groups on the membrane surface. The temperature used in this experiment was 200 °C, and the membrane morphology remained intact after the heat treatment (Figure 3c). However, the color of membranes changed from white to yellow, indicating a certain change in the surface physicochemical properties, which may affect the separation property.

Pore size screening is an important mechanism for membrane separation. The BET [42,43] method was used to detect the pore size distribution and surface area of the membranes. As shown in Figure 3b, the average pore size of the PAN membrane was approximately 35.1 nm, and the distribution range was 1.4–79.6 nm. After modification, the average pore size of the H-PAN membrane decreased to 17.1 nm, and the distribution range narrowed to 2.5–37.9 nm. When heated, the polymer material expands. Therefore, the expansion leads to wrinkles on the membrane surface and compression of the membrane pore size, thereby reducing the overall pore size. As reported, the narrower distribution of membrane pore sizes and smaller membrane pore sizes enabled better interception [44].

**Figure 4:**
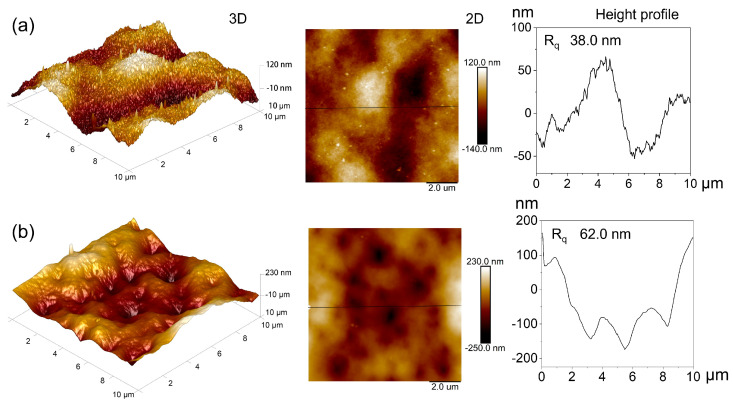
The 3D and 2D AFM images and the corresponding height profile graphs of PAN (**a**) and H-PAN (**b**) membranes.

The SEM images of the PAN and H-PAN membranes are shown in Figure 3d–g and Figure S1. After heat treatment, there were no obvious defects such as fracture or collapse on the surface (Figure 3d–g) of the H-PAN membrane. Compared to the PAN membrane, the surface pore size of the H-PAN membrane was significantly reduced, and the same result can also be seen from the cross sections (Figure S1). This indicated that heat treatment reduced the pore size, which was consistent with the BET results. The AFM images (Figure 4) show the surface roughness of the PAN and H-PAN membranes. The R_q_ represents the root mean square value, which is proportional to the surface roughness [45]. The R_q_ of the H-PAN membrane was 62.0 nm, which was higher than that of the PAN membrane (38.0 nm). The 3D AFM images and the corresponding height profile graphs indicated that the surface of the H-PAN membrane was more uneven than that of the PAN membrane.

**Figure 5:**
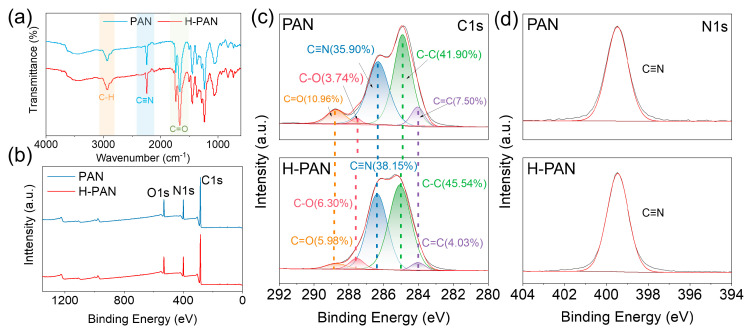
(**a**) FTIR spectra of the PAN and H-PAN membranes. (**b**) XPS spectra of PAN and H-PAN membranes; XPS C1s (**c**) and N1 (**d**) spectra of PAN and H-PAN membranes.

FTIR spectroscopy was used to analyze the functional groups in the PAN and H-PAN membranes [46,47]. As shown in Figure 5a, the structural changes caused by the heat treatment were not significant. Both membranes showed the characteristic peaks for PAN: C≡N stretching vibration and C–H stretching vibration were observed at 2244 cm^−1^ and 3448 cm^−1^, respectively [48]. Additionally, the C=O stretching vibration at 1667 cm^−1^ may be derived from the hydrophilic polymer during the PAN synthesis [49].

Figure 5b shows the XPS spectra of the PAN and H-PAN membranes, while Table 3 shows the changes occurring in the elemental contents. The O element may come from the hydrophilic polymer used in the PAN synthesis. Compared with the PAN membrane, the contents of C and O in the H-PAN membrane were decreased, and the contents of N increased, which was presumably due to the removal of oxygen-containing groups during the heat treatment. By fitting C1s and N1s peaks (Figure 5c,d), the existing form of elements can be identified [50]. The C1s spectrum (Figure 5c) showed a decrease in the C=O content and an increase in the C–O content. This may be caused by the decomposition of C=O groups during annealing. In addition, the C=C content decreased while the C–C content increased, which may be due to the influence of high-temperature treatment, causing the double bond to transition to a more stable single bond. In summary, the main structure of H-PAN did not change significantly.

### 3.2. Wetting Behavior

**Figure 6:**
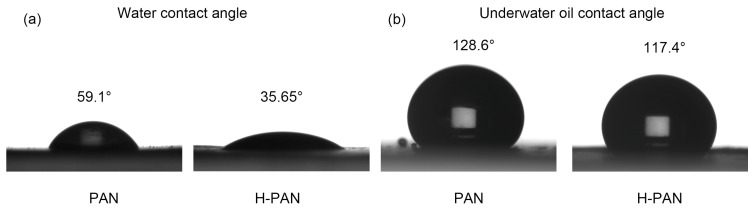
Contact angles of PAN and H-PAN membranes. (**a**) Water contact angle; (**b**) underwater oil contact angle.

Contact angle measurements were conducted to evaluate the membrane surface hydrophilicity [51,52]. The water contact angles were measured in air, and the results are shown in Figure 6a and Figure S2. As shown in Figure 6a, the contact angles of PAN and H-PAN were both less than 90°, indicating that they are both hydrophilic membranes. Compared with the PAN membrane, the water contact angle of the H-PAN membrane was lower, indicating that the hydrophilicity of the H-PAN membrane was higher than that of the PAN membrane. The water contact angle of H-PAN rapidly decreased in the first two seconds and finally stabilized at 35.65°, which was much lower than 59.1° of the PAN membrane (Figure S2). Although some hydrophilic oxygen-containing groups decomposed during the heat treatment process, it also led to an increase in the content of polar group nitrile (-CN), which may be the main reason for the increase in the hydrophilicity of H-PAN. The underwater oil contact angles are shown in Figure 6b; due to the strong oleophobic of the membrane surface underwater, the dynamic contact angle change was difficult to measure, thus a stable contact angle was selected. The underwater oil contact angles of the PAN and H-PAN membranes were 128.6° and 117.4°, respectively. Both PAN and H-PAN membranes exhibited good underwater oleophobic performance.

### 3.3. Nutmeg Oil-in-Water Emulsion Separation

**Figure 7:**
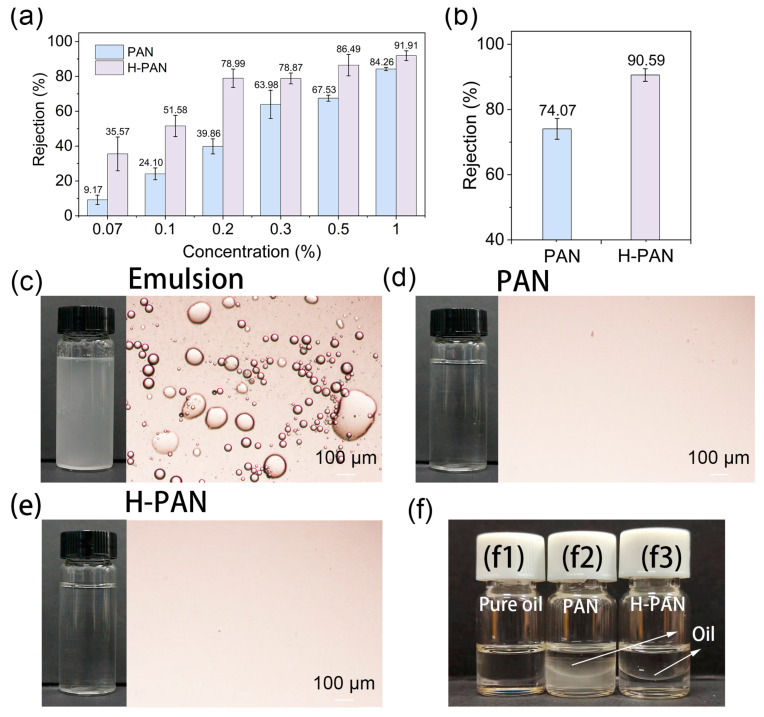
The rejection rates of essential oil in nutmeg essential oil-in-water emulsions of PAN and H-PAN membranes: simulated (**a**) and actual (**b**) emulsion; photographs and optical microscopy images of nutmeg essential oil-in-water emulsion before and after filtration (**c**–**e**); (**f**) photographs of (**f1**) the membrane intercepted liquid of (**f2**) PAN and (**f3**) H-PAN membranes.

To investigate the applicability of the PAN and H-PAN membranes for enriching essential oils, different concentrations of nutmeg essential oil-in-water emulsions were configured and used to study the separation properties. Due to the decrease in pore size, the flux of the H-PAN membrane was lower than that of the PAN membrane (Figure S3). As shown in Figure 7a,b, for both membranes, the oil rejection rate increased with the increase of the EON concentration, and the rejection rates of the H-PAN membrane were always higher than those of the PAN membrane. Compared with the PAN membrane, the H-PAN membrane improved the oil rejection rates by 26–40% at concentrations of 0.07–0.2%, by 15–19% at concentrations of 0.3–0.5%, and by approximately 8% at a concentration of 1%. The results indicate that the H-PAN membrane has a better enrichment effect on low-concentration essential oil.

Further, the two membranes were used to separate actual nutmeg essential oil-in-water emulsions. The fluxes of pure water and the actual emulsions of nutmeg of PAN and H-PAN membranes are shown in Figure S4a,b, and the COD of emulsions with different concentrations are shown in Figure S4c. According to Figure 7b, the oil rejection rate increased from 74.07% for the PAN membrane to 90.59% for the H-PAN membrane. This indicated that the actual EON concentration may be approximately 0.5%, and this was confirmed by the COD values shown in Figure S4c.

Figure 7c–f shows that milky emulsion became a colorless transparent liquid after the membrane separation. The optical microscope photos show that there were a large number of oil droplets in the emulsion with uneven particle size, ranging from several microns to hundreds of microns. A small number of oil droplets with small particle size can be observed in the permeate of the PAN membrane, while almost no visible oil droplets can be observed in the permeate of the H-PAN membrane. The results indicate that large oil droplets were easily intercepted, while smaller oil droplets with better dispersion were more likely to penetrate the membrane. The difference in the interception rate of the membranes may be caused by the difference in membrane pore size. Figure 7(f1) shows that the EON was a colorless and transparent liquid. Because EON is less dense than water, it formed on the upper layer of water (Figure 7(f2,f3)). Both the PAN-based membranes successfully enriched the EON. In particular, the EON enriched by the H-PAN membrane had a higher clarity than that enriched by the PAN membrane, indicating that the H-PAN membrane has a better separation efficiency.

To study the mechanism of pollution, as shown in Table 4, the blocking model of different systems was analyzed, and the schematic diagrams for the models are provided in Figure S5 [53,54]. The main blocking model for the PAN membrane at all selected concentrations was the filter cake blocking model. On the other hand, the H-PAN membrane underwent a transition from the standard blocking model to the complete blocking model. When the concentration was 0.07–0.2%, the H-PAN membrane exhibited the standard blocking model. After the concentration was increased, large oil droplets were deposited at the membrane pores, causing a complete blocking model.

**Figure 8:**
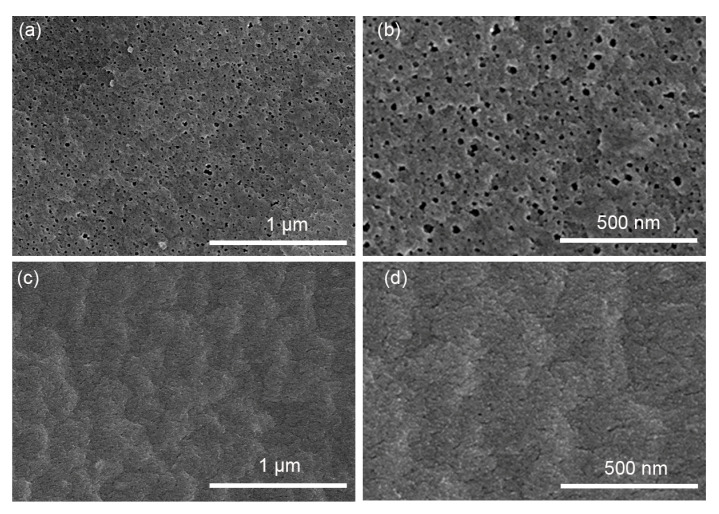
SEM images of the (**a**,**b**) PAN and (**c**,**d**) H-PAN membranes after oil/water separation.

**Figure 9:**
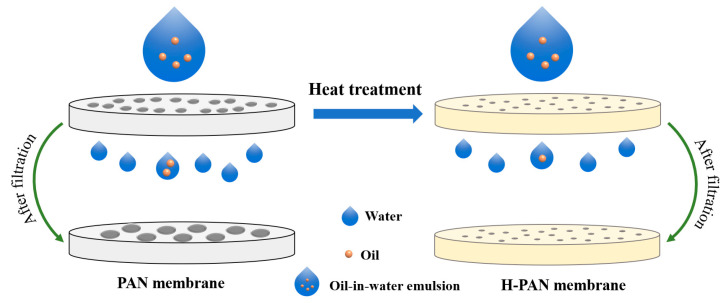
A schematic diagram of the fabrication and emulsion separation processes of the PAN and H-PAN membranes.

To study the stability of the PAN and H-PAN membranes, the membranes after the separation of emulsions were characterized by SEM. As shown in Figure 8a,b, the diameter of the separation pores on the surface of the PAN membrane clearly increased compared to the original membrane. This may be due to the abundant organic components in the EON causing corrosion to the PAN membrane. Figure 8c,d shows that there was no obvious change in the surface morphology of the used H-PAN membrane, indicating that heat treatment could enhance the membrane stability. Figure 9 shows a schematic diagram of the separation of nutmeg essential oil-in-water emulsions with PAN and H-PAN membranes. Overall, H-PAN exhibited a higher oil rejection rate and better stability.

### 3.4. GC-MS Analysis of the Enriched EON

**Figure 10:**
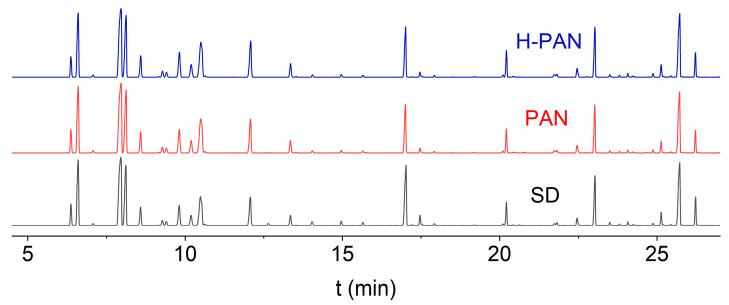
GC-MS results for enriched nutmeg oil.

SD is a traditional method for extracting essential oils and is currently one of the most widely used methods. The components of the EON obtained with SD and the PAN and H-PAN membranes were analyzed with GC-MS. According to Figure 10, the components of the EON enriched by the PAN and H-PAN membranes were similar to those extracted with SD, with similarities of 0.988 and 0.990 (Table 5), respectively. This meant that the composition of the EON was preserved effectively with membrane technology. The main components and relative contents of the EON are shown in Table 6. The main components were terpene compounds, such as bicyclo[2.2.1]heptane, 7,7-dimethyl-2-methylene-; myristicine; 3-Cyclohexen-1-ol, 4-methyl-1-(1-methylethyl)-; α-Pinene; (+)-Camphene; (R)-1-Methyl-5-(1-methylvinyl) cyclohexene; methyleugenol; γ-Terpinene; (+)-4-Carene; elemicine; β-Myrcene; α-Thujene; and safrole. The detection of 53 components further proves that EON components are complex and difficult to separate, and membrane separation could be used for the enrichment of essential oils.

### 3.5. The Applicability of the PAN and H-PAN Membranes for Other TCM Essential Oil-in-Water Emulsions

**Figure 11:**
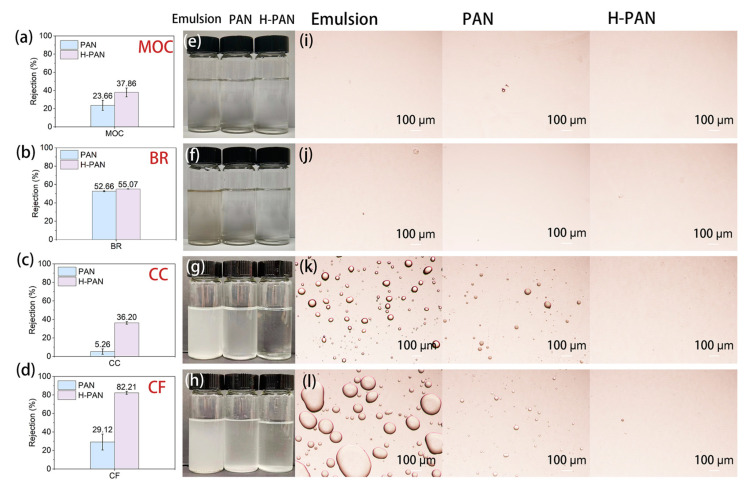
The oil rejection rates of (**a**) MOC, (**b**) BR, (**c**) CC, and (**d**) CF. Photographs of the emulsion permeate through PAN and H-PAN membranes of (**e**) MOC, (**f**) BR, (**g**) CC, and (**h**) CF. The optical microscopy images of the emulsion permeates through PAN and H-PAN membranes of (**i**) MOC, (**j**) BR, (**k**) CC, and (**l**) CF.

To investigate the use of the PAN-based membranes for the enrichment of other essential oils from TCM, the essential oil-in-water emulsions of BR, MOC, CF, and CC were also selected as research subjects. Figure S6a,b shows the fluxes of the four TCM essential oil-in-water emulsions. For the PAN membrane, the fluxes decreased in the order MOC > BR > CF > CC. However, for H-PAN, the difference in flux sizes was not significant. Figure S6c shows the COD contents of the essential oil-in-water emulsions of these four different medicinal herbs; the oil content increased in the order MOC < BR < CC < CF. The separation and enrichment of other essential oils by the PAN-based membrane differed from those of EON. Although CF had the highest oil content (much higher than the 1% nutmeg essential oil-in-water emulsion), its oil rejection rate increased by approximately 53.09% compared to the PAN membrane, which was higher than the 7.65% increase observed for nutmeg essential oil (Figure 11). The oil content of BR was similar to that of the 0.2% concentration of the nutmeg essential oil-in-water emulsion, but unlike the 39.13% increase of nutmeg, the increase of the H-PAN membrane retention rate was not significant.

Figure 11 shows photos and microscopic images of the four types of emulsions and permeates. The photos show that the transparency of the emulsion gradually decreased with the increase in oil concentration. For the MOC with the lowest oil content, there was no significant difference in the permeability of the two membranes. Both membranes can increase the clarity of BR permeate, but the separation difference between the two membranes was not obvious. For CC and CF with relatively high oil content, the clarity of the permeate of the H-PAN membrane was higher than that of the PAN membrane. The microscopic images show that there were obvious oil droplets in the permeate of the PAN membrane, while the oil droplets in the permeate of the H-PAN membrane were reduced. These phenomena indicated that the separation mechanisms for the PAN-based membranes to separate different types of essential oil-in-water emulsions were not completely consistent, and further exploration is needed.

**Figure 12:**
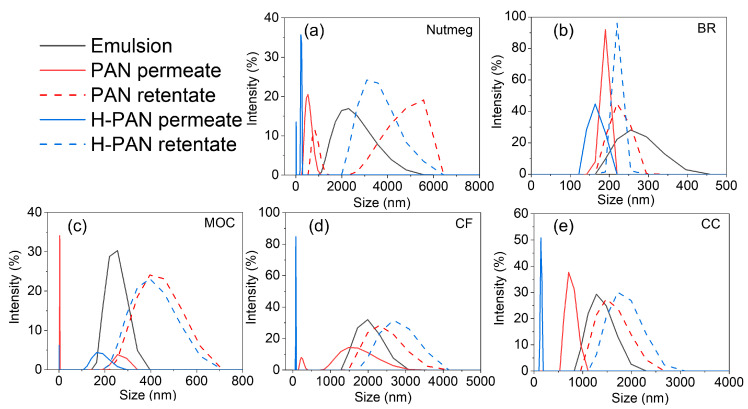
The size distributions of the initial emulsion, permeate, and retentate of nutmeg (**a**), BR (**b**), MOC (**c**), CF (**d**), and CC (**e**).

As shown in Figure 12, the order of the droplet size of different samples was nutmeg > CF > CC > BR > MOC, which was slightly different from the COD results. The order of COD values was CF > CC > nutmeg > BR > MOC (Figure S6c). It was found that although the COD content of CF and CC was higher than nutmeg, the droplet sizes of emulsions were lower than that of nutmeg. This led to a lower oil rejection by CF and CC. It can be inferred that the droplet size was the main factor affecting the oil rejection rate in the separation process. After membrane separation, the droplet sizes of permeates were lower than those of emulsions. Meanwhile, the droplet sizes of the H-PAN membrane permeate were lower than that of the PAN membrane, indicating that the H-PAN membrane could intercept smaller oil drops. From the droplet size distribution of the retentate, was found that the droplet sizes of the retentate of nutmeg, MOC, CF, and CC were all larger than those of the original emulsion, indicating that the oil droplets on one side of the retentate were aggregated with the increasing concentration during membrane separation. However, there was no significant change in the droplet size in the retentate of BR, which may be related to the low oil content of BR.

## 4. Conclusions

In this work, the H-PAN membrane with a smaller pore size and narrower pore size distribution was fabricated through heat treatment. Compared with the PAN membrane, the H-PAN membrane exhibited higher oil rejection rates at different oil contents. Moreover, the morphology of the separated H-PAN membrane was intact, while the pore size of the PAN membrane clearly increased, indicating that H-PAN has better stability for essential oil filtration. Based on the GC-MS data, the similarities of the essential oils enriched by the PAN and H-PAN membranes with those obtained through SD were 0.988 and 0.990, respectively. This indicated that the PAN-based membranes enriched the nutmeg essential oil almost without destructiveness. Moreover, the H-PAN membrane also exhibited a better ability to enrich the essential oils in the BR, MOC, CF, and CC oil-in-water emulsions. In summary, this study provides a design approach for obtaining organic membranes with nanoscale pore size and high stability and preliminarily demonstrates the feasibility of organic membrane enrichment of essential oils.

## Figures and Tables

**Table 1:** Four empirical exponential models used in the experiment.

Model	Equations ^a^
Complete blocking model	$lnJ=lnJ_{0}+K_{c}t$
Intermediate blocking model	$\frac{1}{J}=\frac{1}{J_{0}}+K_{i}t$
Cake filtration model	$\frac{1}{J^{2}}=\frac{1}{J_{0}^{2}}+K_{CA}t$
Standard blocking model	$\frac{1}{J^{0.5}}=\frac{1}{J_{0}^{0.5}}+K_{s}t$

^a^ *J*_0_ is the certain permeate flux when *t* = 0, *J* is the flux (Equation (1)), and *K_c_*, *K_i_*, *K_CA_*, and *K_s_* are the constants for different models.

**Table 2:** The temperamental heating program of GC-MS

Heating Rate (°C/min)	Temperature (°C)	Hold Time (min)
	40	
5	70	5
6	160	5
6	200	0

**Table 3:** Element content of the PAN and H-PAN membranes.

Membrane	Atomic Content (At.%)		
	C/%	N/%	O/%
PAN	76.35	9.94	13.71
H-PAN	73.76	15.67	10.57

**Table 4:** The summary of fouling mechanisms for nutmeg essential oil-in-water emulsions.

Sample	PAN		H-PAN	
	The Blocking Model	R^2^	The Blocking Model	R^2^
0.07%	Cake filtration model	0.9894	Standard blocking model	0.9709
0.1%	Cake filtration model	0.9576	Standard blocking model	0.9479
0.2%	Cake filtration model	0.9770	Standard blocking model	0.9416
0.3%	Cake filtration model	0.9846	Complete blocking model	0.8351
0.5%	Cake filtration model	0.9425	Complete blocking model	0.7916
1%	Cake filtration model	0.8931	Complete blocking model	0.7851
Actual emulsion	Cake filtration model	0.7984	Complete blocking model	0.8432

**Table 5:** The similarities of enriched nutmeg oil.

Sample	Similarity
SD	1.000
PAN	0.988
H-PAN	0.990

**Table 6:** The chemical composition analysis of enriched nutmeg oil.

Number	RT/min	Name	CAS	Formula	Relative Amounts/%		
					SD	PAN	H-PAN
1	6.366	α-Thujene	2867-05-2	C_10_H_16_	2.33	2.52	2.18
2	6.598	α-Pinene	80-56-8	C_10_H_16_	9.28	9.63	8.93
3	7.073	Camphene	79-92-5	C_10_H_16_	0.35	0.32	0.27
4	7.955	Bicyclo[2.2.1]heptane, 7,7-dimethyl-2-methylene-	471-84-1	C_10_H_16_	15.53	16.84	15.54
5	8.113	(+)-Camphene	5794-03-6	C_10_H_16_	8.97	9.75	9.27
6	8.58	β-Myrcene	123-35-3	C_10_H_16_	2.42	2.78	2.73
7	9.274	α-Phellandrene	99-83-2	C_10_H_16_	0.74	0.84	0.85
8	9.4	3-Carene	13466-78-9	C_10_H_16_	0.62	0.71	0.69
9	9.81	(+)-4-Carene	29050-33-7	C_10_H_16_	3.11	3.67	3.83
10	10.185	o-Cymene	527-84-4	C_10_H_14_	1.7	2.06	2.08
11	10.485	(R)-1-Methyl-5-(1-methylvinyl) cyclohexene	1461-27-4	C_10_H_16_	7.12	8.63	8.69
12	10.622	Eucalyptol	470-82-6	C_10_H_18_O	0.3	0.28	0.26
13	12.071	γ-Terpinene	99-85-4	C_10_H_16_	4.38	5.3	5.75
14	12.641	trans-4-Thujanol	17699-16-0	C_10_H_18_O	0.36	——	——
15	13.348	Cyclohexene, 3-methyl-6-(1-methylethylidene)-	586-63-0	C_10_H_16_	1.35	1.62	1.75
16	13.523	α, p-Dimethylstyrene	1195-32-0	C_10_H_12_	0.07	0.08	0.09
17	14.036	4-Thujanol	546-79-2	C_10_H_18_O	0.64	0.36	0.34
18	14.961	2-Cyclohexen-1-ol, 1-methyl-4-(1-methylethyl)-, cis-	29803-82-5	C_10_H_18_O	0.58	0.37	0.3
19	15.649	2-Cyclohexen-1-ol, 1-methyl-4-(1-methylethyl)-, trans-	29803-81-4	C_10_H_18_O	0.44	0.23	0.3
20	16.67	endo-Borneol	507-70-0	C_10_H_18_O	0.06	0.04	0.03
21	17.022	3-Cyclohexen-1-ol, 4-methyl-1-(1-methylethyl)-, (R)-	20126-76-5	C_10_H_18_O	9.44	6.26	6.5
22	17.204	p-Cymen-8-ol	1197-01-9	C_10_H_14_O	0.12	0.06	0.06
23	17.467	α-Terpineol	98-55-5	C_10_H_18_O	1.08	0.53	0.54
24	17.524	2-Cyclohexen-1-ol, 3-methyl-6-(1-methylethyl)-, trans-	16721-39-4	C_10_H_18_O	0.13	0.11	0.12
25	17.924	2-Cyclohexen-1-ol, 3-methyl-6-(1-methylethyl)-, cis-	16721-38-3	C_10_H_18_O	0.22	0.22	0.2
26	19.194	Nerol	106-25-2	C_10_H_18_O	0.09	0.07	0.07
27	20.104	Bornyl acetate	76-49-3	C_12_H_20_O_2_	0.19	0.23	0.25
28	20.215	Safrole	94-59-7	C_10_H_10_O_2_	2.3	2.37	2.61
29	20.427	2-Cyclohexen-1-ol, 3-methyl-6-(1-methylethyl)-, acetate	1204-30-4	C_12_H_20_O_2_	0.06	0.11	0.11
30	20.641	Allyl 4-(2-hydroxyethoxy) benzoate	142651-41-0	C_12_H_14_O_4_	0.07	0.04	0.05
31	21.75	α-Cubebene	17699-14-8	C_15_H_24_	0.3	0.36	0.38
32	21.826	Chavibetol	501-19-9	C_10_H_12_O_2_	0.35	0.33	0.37
33	22.459	Copaene	3856-25-5	C_15_H_24_	0.83	0.9	0.99
34	22.75	β-Copaene	18252-44-3	C_15_H_24_	0.07	0.07	0.08
35	23.024	Methyleugenol	93-15-2	C_11_H_14_O_2_	5.86	5.3	5.76
36	23.495	Caryophyllene	87-44-5	C_15_H_24_	0.32	0.21	0.24
37	23.803	cis-α-Bergamotene	18252-46-5	C_15_H_24_	0.14	0.16	0.18
38	24.069	trans-Isoeugenol	5932-68-3	C_10_H_12_O_2_	0.34	0.28	0.33
39	24.224	(E)-β-Farnesene	18794-84-8	C_15_H_24_	0.09	0.1	0.12
40	24.305	1,4,7-Cycloundecatriene, 1,5,9,9-tetramethyl-, (1Z,4Z,7Z)-	400822-79-9	C_15_H_24_	0.05	0.03	0.04
41	24.873	β-Cubebene	13744-15-5	C_15_H_24_	0.22	0.29	0.33
42	25.131	Isohomogenol	93-16-3	C_11_H_14_O_2_	1.33	1.14	1.26
43	25.194	(+)-Bicyclogermacrene	24703-35-3	C_15_H_24_	0.07	0.07	0.07
44	25.439	β-Bisabolene	495-61-4	C_15_H_24_	0.11	0.12	0.13
45	25.721	Myristicine	607-91-0	C_11_H_12_O_3_	10.37	9.67	10.48
46	25.791	β-Sesquiphellandrene	20307-83-9	C_15_H_24_	0.05	0.05	0.06
47	26.225	Elemicine	487-11-6	C_12_H_16_O_3_	2.68	2.07	2.26
48	26.303	Elemol	639-99-6	C_15_H_26_O	0.06	0.05	0.05
49	26.555	Dodecanoic acid	143-07-7	C_12_H_24_O_2_	0.05	0.04	0.04
50	27.325	Phenol, 2,6-dimethoxy-4-(2-propenyl)-	6627-88-9	C_11_H_14_O_3_	0.05	0.04	0.05
51	27.45	α-Guaiol	489-86-1	C_15_H_26_O	0.05	0.04	0.04
52	28.705	Isoelemicin	5273-85-8	C_12_H_16_O_3_	0.06	0.06	0.07
53	32.765	Myristic acid	544-63-8	C_14_H_28_O_2_	0.35	0.2	0.24

## Data Availability

Data are contained within the article and Supplementary Materials.

## Supplementary Materials

The following supporting information can be downloaded at: https://www.mdpi.com/article/10.3390/membranes14050097/s1. Figure S1: The SEM cross sections of (a,b) PAN and (c,d) H-PAN membranes; Figure S2: Dynamic water contact angle of PAN and H-PAN membranes; Figure S3: The permeation flux variations of different concentrations of nutmeg essential oil-in-water emulsion on (a) PAN and (b) H-PAN membranes; Figure S4: The permeation flux variations of water and nutmeg essential oil-in-water emulsions on (a) PAN and (b) H-PAN membranes; (c) the COD of different concentrations of nutmeg essential oil-in-water emulsions. Figure S5: The schematic diagram of the Hermia model: (a) complete blocking model; (b) standard blocking model; (c) cake blocking model; (d) intermediate blocking model; Figure S6: The permeation flux variations of BR, MOC, CF, and CC essential oil-in-water emulsions on (a) PAN and (b) H-PAN membranes; (c) the COD of BR, MOC, CF and CC essential oil-in-water emulsions.
