# Supplementary material for: Enrichment of Nutmeg Essential Oil from Oil-in-Water Emulsions with PAN-Based Membranes
Source: Membranes (Basel). 2024 Apr 25;14(5):97. doi: 10.3390/membranes14050097 (PMC11122826; doi:10.3390/membranes14050097)
Supplement: Supplementary file 1 [file membranes-14-00097-s001.zip › membranes-2895296-supplementary.pdf]

Supplementary material

Enrichment of Nutmeg Essential Oil from Oil-in-Water Emulsions with PAN-Based Membranes

Huilan Yin [1], Haoyu Zhang [1], Jiaoyang Cui [1], Qianlian Wu [1], Linlin Huang [1], Jiaoyue Qiu [1], Xin Zhang [1], Yanyu Xiang [1], Bo Li [1,2], Hongbo Liu [3], Zhishu Tang [3], Yue Zhang [1,*] and Huaxu Zhu [1,*]

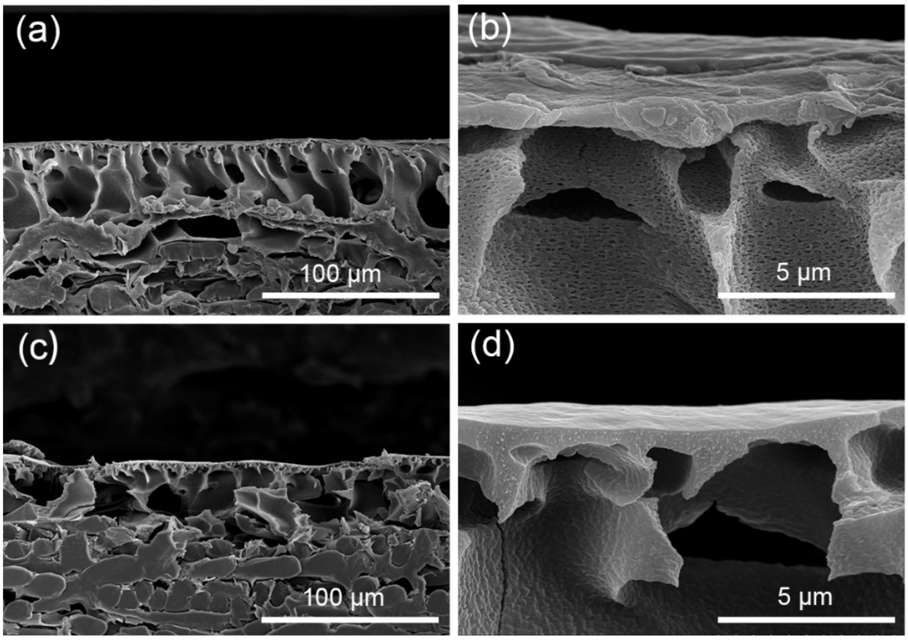

Figure S1. The SEM cross sections of (a, b) PAN and (c, d) H-PAN membranes.

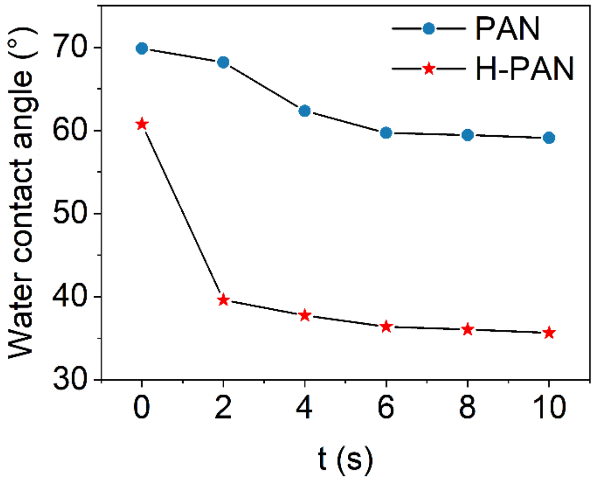

Figure S2. Dynamic water contact angle of PAN and H-PAN membranes.

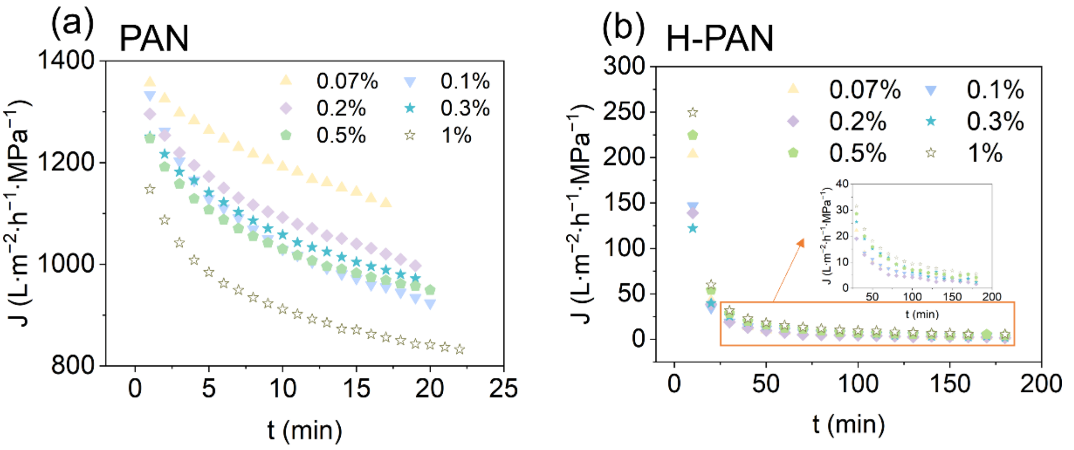

Figure S3. The permeation flux variations of different concentrations of nutmeg essential oil-in-water emulsion on (a) PAN and (b) H-PAN membranes.

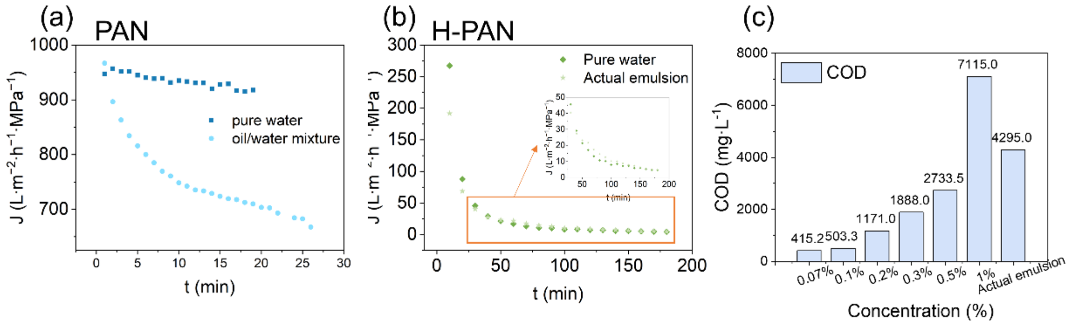

Figure S4. The permeation flux variations of water and nutmeg essential oil-in-water emulsions on (a) PAN and (b) H-PAN membranes; (c) The COD of different concentrations of nutmeg essential oil-in-water emulsions.

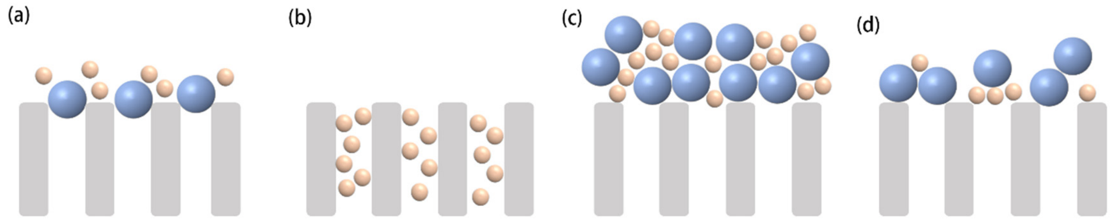

Figure S5. The schematic diagram of the Hermia model. (a) complete blocking model; (b) standard blocking model; (c) cake blocking model; (d) intermediate blocking model.

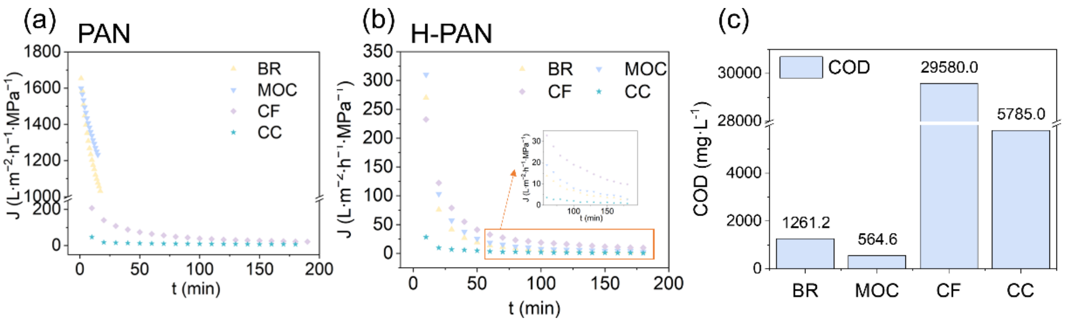

Figure S6. The permeation flux variations of BR, MOC, CF and CC essential oil-in-water emulsions on (a) PAN and (b) H-PAN membranes; (c) The COD of BR, MOC, CF and CC essential oil-in-water emulsions.
